# The impact of shared decision making on transitional preparation in children with recurrent pneumonia

**DOI:** 10.3389/fmed.2025.1628263

**Published:** 2025-09-17

**Authors:** Fan Zhang, Nan Li, Yang Kang, Xing Wang, Yanru Zhao

**Affiliations:** Department of Pediatrics, The Third Affiliated Hospital of Qiqihar Medical University, Qiqihar, Heilongjiang, China

**Keywords:** decision making, shared, precision medicine, pneumonia, patient care management, child, quality of life

## Abstract

**Objective:**

This study evaluates the impact of Shared Decision-Making (SDM) on transitional preparation in children with recurrent pneumonia, highlighting its relevance to precision medicine.

**Methods:**

This retrospective cohort study conducted following the Strengthening the Reporting of Observational Studies in Epidemiology reporting guideline, included 124 children with recurrent pneumonia who were hospitalized between January 2023 and February 2025. Participants were divided into two groups based on the treatment they received: a control group that underwent standard nursing care and an observation group that received SDM interventions. Transition readiness and quality of life (measured by PedsQL 4.0) were assessed and compared between the two groups at three intervals: pre-intervention, 1 month post-discharge, and 3 months post-discharge.

**Results:**

A total of 120 children completed the study, with 60 in each group. Transition readiness improved significantly over time, with main effects for time (F_time_ = 147.329, *P* < 0.05), group (F_group_ = 15.384, *P* < 0.05), and a significant interaction effect (F_interaction_ = 7.338, *P* < 0.05). The observation group consistently demonstrated higher readiness levels. PedsQL4.0 scores showed similar trends (F_time_ = 112.387, *P* < 0.05; F_group_ = 10.842, *P* < 0.05; F_interaction_ = 8.623, *P* < 0.05), favoring the observation group.

**Conclusion:**

Shared Decision-Making enhances transition readiness and quality of life in children with recurrent pneumonia, highlighting its value in precision medicine. These findings support the need for longitudinal studies, SDM training in pediatric education, and policy guidelines to promote SDM, fostering patient-centered care.

## 1 Introduction

Pneumonia remains a leading cause of morbidity and mortality among children globally, claiming over 4 million lives annually in developing nations, with mortality rates ranging from 2.58% to 6.70% among pediatric patients in China (1–3). Statistical data indicates that pneumonia accounts for approximately 24.5%–56.2% of pediatric hospitalizations in China, with a recurrence rate of approximately 7.7%–9.0% (4). Recurrent pneumonia (RP) is characterized by the occurrence of pneumonia two or more times within a 12-month period or a total of three or more episodes, each of which must resolve completely, both clinically and radiologically, according to established criteria for clinical cure (5).

Due to the immaturity of their respiratory and immune systems, children are particularly susceptible to recurrent pneumonia. This susceptibility varies based on multiple factors, including age, health conditions, and socioeconomic environments (6). Premature infants and young children are more vulnerable due to physiological immaturity compared to older children. Children with chronic diseases have higher recurrence rates and complication incidences. In low- and middle-income settings, children often face issues such as indoor air pollution, overcrowding, and malnutrition, and they have limited access to adequate healthcare and vaccinations. Vaccination is a key modifiable risk factor; unvaccinated children face higher risks of initial and recurrent infections. Recurrent pneumonia not only affects respiratory function but can also lead to damage to the nervous and immune systems, impacting growth and development, and even posing life-threatening risks. It results in delayed recovery, reduced quality of life, and significant strain on healthcare resources. In severe instances, RP can pose life-threatening risks, resulting in delayed recovery, diminished quality of life for affected families, and significant strain on healthcare resources (7).

Transition Readiness (TR) refers to the collective capacity of patients, caregivers, and healthcare providers to effectively prepare for, initiate, maintain, and complete the process of transitioning care (8). This encompasses self-care abilities, autonomy in treatment decision-making, and responsibility for health management, including knowledge acquisition, information gathering, and self-management practices. Despite the critical importance of TR, many children diagnosed with pneumonia have not received professional transitional services, resulting in inadequate preparation for care transitions, suboptimal compliance, increased consultation rates, and a decline in quality of life (9).

The cornerstone of high-quality care for children and their caregivers lies in empowering patients to actively participate in their treatment and recovery. This empowerment promotes autonomy in decision-making and encourages engagement in clinical care choices. The Shared Decision-Making (SDM) model represents a patient-centered approach to medical decision-making, wherein patients become integral members of the healthcare team (10, 11). SDM emphasizes respect for patients’ preferences, values, and social contexts while leveraging healthcare professionals’ expertise and evidence-based knowledge to facilitate informed decision-making. Through collaborative discussions that consider patients’ medical preferences and backgrounds, healthcare providers and patients can arrive at optimal medical decisions that ensure high-quality care. The approach enhances patients’ understanding of their conditions, fosters proactive involvement in decision-making, and increases adherence to treatment plans. Ultimately, rehabilitation plans developed through SDM can lead to improved patient outcomes and enhanced quality of life (12).

However, despite the recognized benefits of SDM in various clinical settings, its application in pediatric care, particularly for children with RP, remains underexplored. It is necessary to investigate whether and how SDM interventions can improve TR and quality of life in these vulnerable populations. In this retrospective study, we analyzed data from children with recurrent pneumonia and their parents to evaluate the impact of an SDM intervention program on the children’s readiness for transition and the quality of life of both the children and their caregivers. The study highlights the importance of integrating SDM into pediatric care as a vital component of precision medicine, aiming to tailor healthcare strategies to the unique needs of each patient.

## 2 Materials and methods

### 2.1 Patients

This retrospective cohort study was conducted in accordance with the STROBE (Strengthening the Reporting of Observational Studies in Epidemiology) reporting guidelines to ensure comprehensive and transparent reporting. Previous research has indicated that SDM can improve patient outcomes and quality of life. However, there is limited evidence regarding its effectiveness in pediatric care, particularly for children with recurrent pneumonia. Given the high incidence of recurrent pneumonia among young children, this study aims to explore how SDM can enhance transition readiness and quality of life in this population, thereby addressing the existing research gap.

As a retrospective study, the sample size was determined by the number of eligible patients who met the inclusion and exclusion criteria during the study period from January 2023 to February 2025. During this period, a total of 124 children diagnosed with recurrent pneumonia and admitted to the pediatric department of our hospital were included in the study. Ethical approval was obtained from the Medical Ethics Committee of our hospital (Approval No. 2024LL-30). Written informed consent was provided by the parents or legal guardians of all participating children, who voluntarily agreed to take part in the study.

Additionally, in accordance with best practices for research involving children and adolescents, we paid particular attention to the assent process. We used age-appropriate language to ask each child if they were willing to participate in the study, in the presence of their parents or legal guardians. If the child indicated a willingness to participate, we documented their verbal assent. We confirmed that all children participating in the study had provided assent. For younger children, who may not have fully developed understanding capabilities, we still introduced the basic information about the study in simple and understandable terms, ensuring that they felt comfortable and safe throughout the process.

Inclusion criteria were:

1. Children aged between 3 and 14 years old.
2. The predominant illness among children is pneumonia.
3. Children presenting pneumonia occurring at least twice within a 1-year period, or those experiencing pneumonia on three or more occasions.
4. Children with 2 pneumonia intervals of more than 7 days.

Exclusion criteria were:

1. Children with comorbidities such as impaired organ function or respiratory diseases including bronchial asthma and bronchodilatation.
2. Children presenting with more than 10 days of illness or exhibiting clinical characteristics consistent with lobar pneumonia or interstitial pneumonia.
3. Children who have received immunoglobulin, hormones, or other immunosuppressive drugs within the past 3 months, or those with a history of surgical trauma within the past 3 months.

In this retrospective study, the 124 children with recurrent pneumonia were categorized into two groups based on the treatment they received: a control group (62 cases) that underwent standard nursing care and an observation group (62 cases) that received SDM interventions. However, due to transfers to the NICU and loss to follow-up, 2 children from each group were excluded. Ultimately, 120 children completed the study, comprising 60 participants in the control group and 60 participants in the observation group.

### 2.2 Intervention methods

#### 2.2.1 Intervention methods of the control group

The control group implements standard nursing procedures, which encompass alleviating symptoms such as fever, cough, phlegm, fatigue, and sleep disorders. This includes providing guidance to children on the proper administration of anti-inflammatory drugs, cough suppressants, expectorants, and emphasizing the importance of monitoring their effectiveness and potential side effects. Additionally, it focuses on delivering comprehensive care in areas such as oxygen therapy, dietary management, sleep regulation, psychological support.

#### 2.2.2 Intervention methods of the observation group

The observation group implemented the SDM intervention process based on the routine diagnosis and treatment of the control group.

##### 2.2.2.1 Establishing an intervention team and developing a decision-making roster

The SDM intervention team comprises of a postgraduate pediatric chief physician with 18 years of experience in pediatrics, two postgraduate pediatric attending physicians with 5 years of experience in pediatrics, a rehabilitation instructor with 6 years of expertise in rehabilitation, an undergraduate pediatric nursing administrator with 20 years of experience in pediatrics, two undergraduate pediatric nurses with 6 years of experience in pediatrics, and the researcher responsible for the development and implementation of a SDM program for children suffering from recurrent pneumonia. Prior to the intervention, the researcher provided team members with extensive theoretical training and practical simulations. Theoretical training sessions were held three times weekly, each lasting 2 h. These sessions covered the conceptual framework and semantic subtleties of SDM, the current research landscape, sequential implementation strategies, and their clinical implications. In order to expedite the adaptation process to the new role, team members underwent simulation training subsequent to theoretical instruction, enabling prompt rectification and enhancement of any issues encountered during the simulations.

Based on a comprehensive review of relevant literature from both domestic and international sources, the team members developed various treatment and care methods for children with recurrent pneumonia and created a detailed “Decision Checklist for Children with Recurrent Pneumonia” (hereinafter referred to as the “Decision Checklist”). This checklist outlines specific criteria for selecting medications, including anti-inflammatory drugs, antiviral drugs, cough suppressants, and bronchodilators, as well as guidelines for oxygen therapy administered via nebulization or inhalation.

To ensure the scientific validity of the decision checklist, we adopted stringent inclusion criteria for the literature. The criteria are as follows: This study only included systematic reviews, randomized controlled trials (RCTs), cohort studies, and case-control studies to guarantee high-quality and reliable evidence. Participants were required to be recurrent pneumonia patients aged between 0 and 18 years. Interventions included, but were not limited to, anti-inflammatory drugs, antiviral medications, cough suppressants, bronchodilators, oxygen therapy, nebulizer treatments, transdermal drug delivery, ultraviolet irradiation, ultrashort wave therapy, acupuncture, and cupping. Outcome measures primarily focused on transition readiness and quality of life, aiming to comprehensively assess the efficacy and safety of different treatments for pediatric patients with recurrent pneumonia. Through these rigorous criteria, we strive to provide reliable and clinically relevant decision support.

To ensure the acceptability and applicability of the decision checklist and treatment protocol, they were initially drafted based on a literature review and clinical experience. Multiple experts in pediatrics then reviewed the drafts and provided suggestions for improvement. On this basis, 30 patients and their families were selected for pilot testing. Feedback was collected through one-on-one interviews, focusing on their understanding of their child’s condition, their views on the treatment protocol, and their evaluation of the usability of the decision checklist. This feedback was used to further refine the materials, ensuring they were clear and relevant to patients’ actual situations. After several revisions and confirmations, the final versions of the decision checklist and treatment protocol were approved by all participating experts and underwent a final comprehensive review before the study commenced to ensure their scientific rigor and practicality. Additionally, to emphasize child-centered care, children aged 7–14 were involved in testing the decision checklist’s usability. Healthcare professionals assessed the children’s understanding of treatment options and used their feedback to make the checklist more intuitive and easier to understand, such as by adding illustrations and simple language explanations.

The list includes the principles of anti-inflammatory, antiviral, antitussive, and asthma medications. Additionally, it examines the advantages and disadvantages of various treatment modalities, including oxygen therapy, nebulized inhalation, transdermal drug delivery, ultraviolet light irradiation, ultrashort-wave therapy, acupuncture, and cupping. Furthermore, this document includes information on medication duration, common complications, hospitalization costs, and timeframes. It also addresses the risk of recurrence and the available options for re-treatment following a recurrence.

##### 2.2.2.2 Establishing a collaborative relationship and evaluating the necessity for SDM

After the children were admitted to the hospital, the researcher, supervising physician, and nurse worked together to build a strong rapport with both the children and their parents. Initially, an assessment was conducted to evaluate the child’s current disease status and the parents’ understanding of pneumonia. Following this, a thorough explanation of the available treatment and care tools for managing pneumonia was provided. The various treatment options the child might encounter were also discussed, along with a clear outline of the purpose and process of SDM.

The nurses then delivered tailored health education to both the child and parents regarding recurrent pneumonia, covering etiological factors, treatment alternatives, and daily care practices, including dietary habits, living environment adjustments, and routines for managing recurrent pneumonia. They addressed and corrected parental misconceptions related to medication administration, examinations, treatments, and caregiving, consistently reinforcing this information during regular rounds and conversations.

Medical professionals carefully explained each item on the decision-making checklist to ensure that both children and parents fully understood. They distributed the checklist and allowed ample time for reflection, enabling families to make informed choices tailored to their treatment expectations, personal values, preferences, and family circumstances.

##### 2.2.2.3 Provide decision-making guidance and determine the final decision

Providing decision guidance and determining the final decision involves in-depth communication with children and parents to address questions and resolve any confusion. When facing decision difficulties, appropriate guidance and suggestions are provided to enhance the family’s motivation and autonomy in decision-making. For example, in medication selection, healthcare professionals thoroughly explain the mechanisms of action, potential side effects, and appropriate populations for different anti-inflammatory drugs; in the choice of oxygen therapy, they detail the indications, expected outcomes, and possible complications, and showcase successful cases to boost confidence. The medical team also discusses the specific steps of various treatment pathways, such as the advantages and disadvantages of nebulization or transdermal drug delivery, ensuring that families fully understand each treatment option. Healthcare professionals provide detailed written materials and ensure clear understanding through face-to-face interactions, allowing ample time for reflection and discussion. This enables families to make informed choices based on treatment expectations, personal values, preferences, and family circumstances. The final decision is made jointly by doctors and patients and is continuously evaluated during hospitalization, with adjustments made dynamically according to the child’s physiological and psychological needs and disease progression.

#### 2.2.3 Intervention deployment

This is a retrospective study, and the treatments had already been completed at the time of the study. The healthcare providers who performed the interventions and the data analysts were blinded to whether the patients were included in the study and to their group assignments, thereby reducing potential bias during the process.

To ensure consistent implementation of SDM by healthcare professionals, we developed detailed Standard Operating Procedures (SOPs) and implemented strict quality control measures. We created a comprehensive SDM training manual covering all steps, key points, and considerations to ensure uniform standards. Monthly training sessions keep team members updated on the latest SDM concepts and techniques, with regular assessments of their skills. A dedicated quality monitoring team checks compliance with SOPs, records issues, and proposes improvements. We also established a feedback mechanism involving patients and families, collecting opinions through surveys and interviews to adjust and improve intervention measures, ensuring their effectiveness and acceptability. These measures aim to achieve efficient SDM implementation and continuous improvement across all levels.

### 2.3 Research tools

#### 2.3.1 General information questionnaire for children

Following a comprehensive literature review, team members developed their own questionnaire encompassing variables such as age, gender, frequency of pneumonia occurrences per year, presence of fever and cough symptoms, respiratory rate, primary caregiver information, and monthly family income. To ensure the reliability and validity of the questionnaire, we calculated Cronbach’s Alpha coefficient, which showed that the questionnaire had good internal consistency (Cronbach’s Alpha = 0.804). Additionally, we verified the construct validity of the questionnaire through factor analysis (KMO = 0.795). The results indicated that all indicators of the questionnaire possessed sufficient validity.

#### 2.3.2 Chinese version of self-management and transition to adulthood with Rx = Treatment questionnaire (STARx)

The STARx questionnaire was compiled by scholars Ferris et al. in 2015 (13), which is a self-rating scale for children to assess the level of self-management and transition readiness. The Chinese version of STARx questionnaire was revised by our Ma Jiali et al. (14). The questionnaire comprises four dimensions of medication management (with six items), doctor-patient communication (with five items), health participation (with five items), and knowledge of disease (with two items). In total, there are 18 items, and the Likert’s 5-point scale is used to assign a maximum score of 90 points. A higher score indicates greater self-management ability and transition readiness level. The total Cronbach’s alpha coefficient of the questionnaire was 0.83, and the content validity index was 0.96.

#### 2.3.3 Chinese version of pediatric quality of life inventory (PedsQL 4.0)

PedsQL 4.0 scale was developed by American scholars Varni et al. in 2001 to measure children’s health-related quality of survival (15). The Chinese version of the PedsQL 4.0 scale was revised by Lu Yiyun et al. in China (16). The scale contains 4 dimensions of physical functioning (8 entries), emotional functioning (5 entries), role functioning (5 entries), and social functioning (5 entries), with a total of 23 entries. The study employed a Likert 5-point scale, with a maximum score of 100, where higher scores indicated better quality of life. The internal consistency reliability analysis using Cronbach’s alpha coefficients yielded values ranging from 0.74 to 0.82 for each dimension of the scale.

#### 2.3.4 Chinese version of pediatric quality of life inventory TM family impact module (PedsQL TM FIM)

The PedsQL TM FIM scale was developed by Varni et al. (17) in 2004 to assess the extent of the impact of the child’s illness on the parents’ quality of survival and family functioning. The Chinese version of the PedsQL TM FIM scale was revised by Ruoqing Chen et al. (18) in China, and the scale contains 4 dimensions of physical functioning (6 entries), social functioning (4 entries), emotional functioning (5 entries), and cognitive functioning (5 entries), with a total of 20 entries. A Likert 5-point scale with a total score of 100 was used, with higher scores indicating a higher quality of life for the parents of the child. The total Cronbach’s alpha coefficient of the scale was 0.95, and the Cronbach’s alpha coefficients for each entry ranged from 0.830 to 0.932.

### 2.4 Data collection

The data collection was conducted at three time points: upon admission, 1 month post-discharge, and 3 months post-discharge. The STARx, PedsQL4.0, and PedsQLTM FIM scales were administered after admission, at the 1-month follow-up, and at the 3-month follow-up. To ensure the quality of the data, researchers guided children or their parents in completing the questionnaires, ensuring that all questions were fully answered with no omissions. Responses were required to be logically consistent, reasonable, and aligned with actual circumstances. Additionally, questionnaires had to be completed within the specified time frames. By employing these methods, researchers ensured the validity of the questionnaires during the data collection process.

### 2.5 Statistical methods

SPSS22.0 was used for statistical analysis, continuous variables were described by (±s), categorical variables were described by constituent ratios, *t*-test was used for measurement data, χ^2^ test was used for counting data. Repeated measures ANOVA were used to compare the data of the two groups and analyze the time effect, group effect, and interaction effect. *P* < 0.05 indicates that the difference is statistically significant.

## 3 Results

### 3.1 Comparison of the general data of children in the two groups

The general data of children in the two groups were compared using *t*-test and χ^2^ test, and the differences were not statistically significant (all *P* > 0.05, Table 1).

**TABLE 1:** Comparison of the general data of the two groups of children with RP.

Item	Control group (*n* = 60)	Observation group (*n* = 60)	*t*/χ^2^	*P*
Age	5.20 ± 0.684	5.10 ± 0.630	0.833	0.406
**Gender**				
Male	33 (55.0%)	35 (58.3%)	0.136	0.713
Female	27 (45.0%)	25 (41.7%)		
**Number of pneumonias/year**				
2∼3	46 (76.7%)	41 (68.3%)	1.287	0.525
3∼5	12 (20.0%)	15 (25.0%)		
>5	2 (3.3%)	4 (6.7%)		
**Fever**				
Low-grade fever	17 (28.3%)	17 (28.3%)	1.139	0.768
Medium-grade fever	34 (56.7%)	30 (50.0%)		
Ardent fever	7 (11.7%)	50 (18.3%)		
Ultrahyperpyrexia	2 (3.3%)	23.3%		
**Cough**				
Yes	48 (80.0%)	46 (76.7%)	0.196	0.658
No	12 (20.0%)	14 (23.3%)		
**Breaths per minute**				
20∼30	43 (71.7%)	44 (73.3%)	1.011	0.603
30∼40	15 (25.0%)	12 (20.0%)		
>40	2 (3.3%)	4 (6.7%)		
**Primary caregiver**				
Parents	44 (73.3%)	43 (71.7%)	0.042	0.838
Grandparents	16 (26.7%)	16 (28.3%)		
**Parent age**				
20∼30	17 (28.3%)	17 (28.3%)	0.562	0.967
30∼40	19 (31.7%)	22 (36.7%)		
40∼50	9 (15.0%)	9 (15.0%)		
50∼60	11 (18.3%)	9 (15.0%)		
≥60	4 (6.7%)	3 (5.0%)		
**Education of parents**				
Primary school and below	13 (21.7%)	17 (28.3%)	0.731	0.866
Middle school	19 (31.7%)	18 (30.0%)		
College or undergraduate	18 (30.0%)	16 (26.7%)		
Graduate student or above	10 (16.7%)	9 (15.0%)		
**Residence**				
Cities and towns	26 (43.3%)	28 (46.7%)	0.135	0.714
Countryside	34 (56.7%)	32 (53.3%)		
**Monthly per capita family income (RMB)**				
<3000	17 (28.3%)	20 (33.3%)	0.453	0.798
3000∼5000	23 (38.3%)	20 (33.3%)		
>5000	20 (3.3%)	20 (33.4%)		
**Sources of medical payment**				
Hospitalization insurance	41 (68.3%)	44 (73.3%)	0.363	0.547
One’s own expense	19 (31.7%)	16 (26.7%)		

### 3.2 Comparison of the baseline data of the two groups of children

The *t*-test was used to compare the baseline data of the two groups of children before intervention, and the differences were not statistically significant (all *P* > 0.05, Table 2).

**TABLE 2:** Comparison of the baseline data of the two groups of children before intervention.

Item	Control group (*n* = 60)	Observation group (*n* = 60)	*t*	*P*
STARx	55.43 ± 5.662	54.36 ± 7.105	0.912	0.364
PedsQL4.0	67.16 ± 6.425	66.14 ± 5.274	0.950	0.344
PedsQLTM FIM	70.12 ± 7.086	69.59 ± 5.272	0.465	0.643

### 3.3 Comparison of TR levels between the two groups of children

A two-factor repeated-measures ANOVA was used to compare the STARx levels of the two groups pre-intervention, 1-month post-discharge, and 3-months post-discharge. The results showed that the time main effect was statistically significant (F_time_ = 147.329, *P* < 0.05), i.e., without considering the intervention factor, the level of children’s transition readiness increased over time; the group main effect was statistically significant (F_group_ = 15.384, *P* < 0.05), i.e., without considering the time factor, the level of children’s transition readiness in the observation group differed from that of the control group and the observation group was higher than the control group; the interaction between the group effect and the time effect was statistically significant (F_interaction_ = 7.338, *P* < 0.05), i.e., the trend of the children’s level of transition readiness in the two groups was different in the pre-intervention period, 1 month after discharge, and 3 months after discharge, and the observation group was higher than the control group (Table 3).

**TABLE 3:** Repeated measures ANOvariance of STARx levels in children with RP (score, $\bar{x}$ ± s).

Item	Time	STARx scores	Drug management	Doctor-patient communication	Health participation	Disease knowledge
Control group (*n* = 60)	PI	55.43 ± 5.662	19.67 ± 3.493	14.58 ± 2.271	14.89 ± 3.672	6.29 ± 2.357
	1-month PD	62.68 ± 8.370	21.26 ± 4.748	17.34 ± 2.409	16.98 ± 2.563	7.11 ± 2.490
	3-months PD	70.18 ± 8.103	22.99 ± 3.902	18.69 ± 2.783	20.43 ± 3.251	8.08 ± 1.437
Observation group (*n* = 60)	PI	54.36 ± 7.105	19.13 ± 3.453	14.16 ± 1.895	14.92 ± 2.850	6.16 ± 2.132
	1-month PD	68.37 ± 7.018*	23.67 ± 2.981*	17.99 ± 2.358	20.56 ± 2.117*	7.81 ± 1.430
	3-months PD	83.06 ± 3.236*	27.84 ± 2.011*	22.05 ± 2.002*	23.89 ± 2.093*	9.29 ± 0.641*
*F*_*time*_		289.424	60.662	150.559	108.116	30.845
*F*_*groups*_		28.203	15.266	9.503	35.044	4.984
*F*_*interaction*_		30.209	12.187	15.939	7.105	2.301

PI, pre-intervention; PD, post-discharge;

*In comparison to the control group at the same time point, *P* < 0.05.

### 3.4 Comparison of the quality of survival between the two groups of children

A two-factor repeated-measures ANOVA was used to compare the pre-intervention, 1-month post-discharge, and 3-months post-discharge PedsQL4.0 levels of the two groups of patients. The results showed that the main effect of time was statistically significant (F_time_ = 112.387, *P* < 0.05), regardless of the intervention factors, the quality of life of the children has improved with longer time. The main effect of group was statistically significant (F_groups_ = 10.842, *P* < 0.05), regardless of the time factor, there was a difference in the quality of life between the two groups, and the observation group was higher than the control group. The interaction between the group effect and the time effect was statistical significance (F_interaction_ = 8.623, *P* < 0.05), the groups showed different trends before intervention, 1 month after discharge, and 3 months after discharge, and the observation group were higher than the control group (Table 4).

**TABLE 4:** Comparison of PedsQL4.0 scores between two groups of children with RP ($\bar{x}$ ± s).

Item	Time	PedsQL4.0 scores	Physiologic function	Emotional function	Role function	Social function
Control group (*n* = 60)	PI	67.16 ± 6.425	22.27 ± 4.376	14.74 ± 2.178	15.36 ± 3.183	14.78 ± 3.001
	1-month PD	75.86 ± 4.979	25.21 ± 2.783	16.87 ± 2.394	17.93 ± 2.631	15.83 ± 3.225
	3-months PD	81.31 ± 5.871	27.10 ± 3.594	18.42 ± 2.918	19.76 ± 2.843	16.03 ± 2.639
Observation group (*n* = 60)	PI	66.14 ± 5.274	22.09 ± 3.904	14.56 ± 2.061	15.21 ± 2.906	14.28 ± 2.843
	1-month PD	84.11 ± 6.710*	27.95 ± 3.207*	18.29 ± 1.973*	19.46 ± 3.271*	18.43 ± 2.552*
	3-months PD	89.39 ± 5.583*	28.17 ± 2.648	20.17 ± 2.152*	21.52 ± 2.719*	19.55 ± 3.221*
*F*_time_		191.067	38.287	70.234	61.743	22.841
*F*_groups_		38.185	5.523	9.943	5.972	23.106
*F*_interaction_		14.492	3.478	3.392	2.255	8.560

PI, pre-intervention; PD, post-discharge;

*In comparison to the control group at the same time point, *P* < 0.05.

### 3.5 Comparison of PedsQLTM FIM scores among parents of children with RP

A two-factor repeated-measures ANOVA was used to compare the pre-intervention, 1-month post-discharge, and 3-months post-discharge PedsQLTM FIM levels. The results showed that the time main effect was statistically significant (F _time_ = 136.874, *P* < 0.05), i.e., without considering the intervention factor, the quality of life for families of affected children’s parents improved over time. The group’s main effect was statistically significant (F_group_ = 15.362, *P* < 0.05), i.e., when disregarding the time factor, a significant difference in the quality of life was observed between the families of affected children in the two groups, with the observation group reporting a higher quality of life compared to the control group. The interaction between group effect and time effect was statistically significant (F_interaction_ = 11.028, *P* < 0.05), i.e., the trends in the quality of life for families with children in the two groups differed significantly during the pre-intervention period, 1 month post-discharge, and 3 months post-discharge, with the observation group consistently demonstrating higher levels compared to the control group (Table 5).

**TABLE 5:** Comparison of PedsQLTM FIM scores among parents of children with RP ($\bar{x}$ ± s).

Item	Time	PedsQLTM FIM scores	Physiologic function	Social function	Emotional function	Cognitive function
Control group (*n* = 60)	PI	70.12 ± 7.086	18.55 ± 3.214	16.36 ± 3.621	17.32 ± 3.412	17.89 ± 2.3381
	1-month PD	74.97 ± 5.609	20.34 ± 2.904	17.83 ± 2.904	18.77 ± 3.102	18.04 ± 3.625
	3-months PD	83.85 ± 5.833	22.67 ± 3.912	18.42 ± 3.672	21.01 ± 3.671	21.74 ± 2.887
Observation group (*n* = 60)	PI	69.59 ± 5.272	18.76 ± 3.452	16.10 ± 2.312	17.19 ± 2.672	17.55 ± 3.973
	1-month PD	83.18 ± 6.083*	23.52 ± 2.893*	19.37 ± 3.672*	20.09 ± 3.842*	20.22 ± 2.538*
	3-months PD	92.20 ± 5.550*	26.75 ± 3.002*	19.56 ± 3.124*	22.69 ± 2.945*	22.19 ± 3.141
*F*_time_		151.712	54.470	20.135	35.148	33.451
*F*_groups_		48.068	38.207	5.354	3.962	2.995
*F*_interaction_		11.890	6.050	2.775	1.527	2.959

PI, pre-intervention; PD, post-discharge;

*In comparison to the control group at the same time point, *P* < 0.05.

## 4 Discussion

The results of this study confirm our primary conclusions, demonstrating that the implementation of SDM interventions significantly improved the transition readiness and quality of life for children with recurrent pneumonia.

Studies have shown that inadequate preparation for transition has been demonstrated to be the primary etiology of recurrent pneumonia in children, resulting in non-compliance, disease exacerbation, increased readmission rates, and potential cardiopulmonary failure (19–21). These consequences impose significant physical and psychological burdens on affected children, severely impacting their education and social integration while also profoundly affecting the overall quality of life for their families. Assisting children in preparing for the transition is a pressing concern for caregivers. This study applies a shared decision-making healthcare model to assist in the development and implementation of strategies, thereby improving affected children’s and their parents’ understanding and adherence to treatment protocols, safeguarding their quality of life, and optimizing treatment outcomes.

### 4.1 The implementation of SDM can effectively enhance the TR in children with RP

The data analysis revealed statistically significant differences (*P* < 0.05) in the time effect, between-group effect, and interaction effect when comparing the transition readiness of children in the two groups after the intervention. This finding resonates with the results of studies on children with severe illnesses, indicating that effective training can enhance children’s self-care abilities, parents’ disease management skills, and children’s transition readiness (22). Compared to previous studies, the novelty of this research lies in not only validating the positive role of SDM in acute-phase decision-making but also being the first to demonstrate its long-term positive impact on self-management abilities and transition readiness after discharge in children with RP. These findings indicate that SDM effectively enhances the transition readiness of children with RP. The reason for this may be attributed to the emphasis of SDM on doctor-patient equality, whereby medical personnel elucidate the characteristics, indications, precautions, and factors influencing therapeutic outcomes of diverse diagnostic and treatment strategies. This ensures comprehensive comprehension among patients, thereby facilitating their profound understanding of pneumonia treatment and rehabilitation knowledge (23). At the same time, the children and their parents express their concerns and ideas to the medical personnel, engaging in discussions the most appropriate diagnostic and treatment for their children. Furthermore, they actively participate throughout the entire treatment and care process, enhancing the TR of children with RP. This active involvement fosters self-health promotion among children, improves their sense of health responsibility, and standardizes their health behaviors. The implementation of shared decision-making in this study was mainly through the intervention of medical staff as transition coordinators, using artificial intelligence technology to more accurately and efficiently obtain existing and potentially important information about the child through subjective and objective information, alongside disease education and training for children and caregivers. These interventions aim to provide appropriate transition preparation services, facilitate effective support for children and caregivers, enhance their knowledge and management skills related to the disease, and promote overall readiness for transition.

### 4.2 SDM improves the quality of survival of children and parents with RP

Quality of survival is a key indicator of patient outcomes and satisfaction (24). The results of this study showed that the differences in the time effect, between-group effect and interaction effect were statistically significant (*P* < 0.05) when comparing the quality of survival of children and parents in the two groups after the intervention. This finding is highly consistent with previous research conclusions, demonstrating that SDM combined with personalized care significantly reduces pain in pediatric palliative care patients, shortens hospital stays, and increases family satisfaction (25). It means that SDM can effectively improve the quality of survival of children with recurrent pneumonia and their parents. During the intervention, the medical staff apply artificial skills and technical analysis and overall responsibility for medical professional problems faced by children and their parents (such as medication, treatment, diet, excretion, etc.) to promote the recovery of the physiological state of the children. Moreover, prior to selecting the optimal care plan, the intervention team conducted a comprehensive assessment of the values, preferences, and decision-making requirements of both children and their parents. In addition, they aligned the children’s personality traits with robust clinical evidence to facilitate precise decision-making and alleviate anxiety, helplessness, and other adverse emotions experienced by both parties. Consequently, this approach enhanced satisfaction levels and fostered a sense of fulfillment in the decision-making process for both children and parents. What’s more, the active involvement of parents and effective doctor-patient communication foster trust in medical professionals among children and their parents, thereby enhancing treatment adherence. Also, the establishment of a supportive family system creates favorable conditions for children to reintegrate into society and expedite their adaptation to familial and social roles. This ultimately enhances the subjective well-being of both children and parents, contributing significantly to an improved quality of life. It is important to note that this study excluded children with certain complex health conditions. Although the results indicate that SDM can improve quality of life, these findings primarily apply to specific child populations. Generalizing these conclusions to all children may introduce bias. Future research should explore the effectiveness of SDM in a broader range of children to ensure its general applicability.

### 4.3 Recommendations to improve shared decision making for children with recurrent pneumonia

Studies have shown that more than half of the parents of children want to share decision-making with their doctors (26), but the application of shared decision-making in pediatrics still faces many challenges due to the limited availability of medical resources, shortage of pediatricians, and lack of understanding of shared decision-making (27). In order to give full play to the positive effects of shared decision making in pediatrics, it is recommended that healthcare institutions and medical staff make efforts in some areas. First, enhance the involvement of pediatric nurses and other healthcare professionals. Given their frequent and direct interaction with children and families, nurses are uniquely positioned to play a crucial role in facilitating family decision-making processes. Then, improve the awareness of medical staff and parents of children on shared decision-making. We can expand the publicity of knowledge of pediatric diseases and shared decision-making through hospital training, lectures, admission education, public numbers and distribution of colorful leaflets, so as to promote the acceptance and clinical application of shared decision-making by medical staff and parents of children. Next, further optimize the process of shared decision-making, embed it in daily clinical work, and improve the efficiency of shared decision-making on the basis of not affecting the diagnosis and treatment of children. In the end, apply modern information technology to develop decision-making aids suitable for pediatrics to increase interest while alleviating the conflicts of time and personnel shortage.

### 4.4 Limitations and future perspectives

This study has several limitations that should be acknowledged. Firstly, the sample size was relatively small and limited to a single hospital, which may affect the generalizability of the findings. The single-center design limits the external validity of the results, as different healthcare institutions may exhibit significant variations in patient populations, medical resources, and standards of care. Secondly, the study relied on self-reported measures for assessing transition readiness and quality of life, which may introduce bias. Additionally, the follow-up period was limited to 3 months, which may not capture long-term outcomes of the SDM intervention. It is also important to note that this study excluded children with severe complications or other complex health conditions, who may be more susceptible to recurrent pneumonia. Therefore, although our results indicate that SDM has a significant positive effect on improving quality of life, these findings primarily apply to the children who met our inclusion criteria. Generalizing these conclusions to “all children” may introduce unnecessary bias. Lastly, the study did not account for potential confounding variables such as socioeconomic status and parental education level, which could influence the results. These factors may have a significant impact on patient adherence and treatment outcomes. Future research should focus on expanding the sample size and including multiple centers to enhance the generalizability of the findings. Longitudinal studies with extended follow-up periods are needed to assess the long-term effects of SDM on transition readiness and quality of life. Moreover, incorporating objective measures and controlling potential confounders will strengthen the validity of future studies. Exploring the integration of digital tools and artificial intelligence in SDM could also provide innovative ways to enhance decision-making processes and outcomes in pediatric care.

Considering SDM’s positive impact, exploring its role in achieving Sustainable Development Goals (SDGs) is vital. SDM improves children’s health and quality of life by increasing understanding and adherence to treatments, supporting universal health coverage. It promotes quality education through personalized health education, enhancing disease management and self-care skills. SDM also encourages social inclusion and reduces inequality by respecting individual needs and values. Multidisciplinary teamwork improves healthcare quality and access. Thus, SDM benefits individual patients and contributes to global SDGs. Future work should focus on expanding and refining SDM to benefit more patients and foster societal advancement.

### 4.5 Educational impact

This study provides significant educational insights. Systematic training can enhance healthcare providers’, children’s, and parents’ understanding and application of SDM. Medical institutions should regularly offer SDM training to update healthcare providers on the latest theories and techniques. Hospitals can promote SDM among parents and children through lectures and workshops, particularly by providing personalized health education resources for families of children with recurrent pneumonia. Multidisciplinary team collaboration in SDM facilitates individualized treatment planning and comprehensive support. Developing pediatric-appropriate decision aids, such as mobile apps or online platforms, helps parents and children understand treatment options, document illness progression, and communicate with healthcare providers, thus improving SDM efficiency and engagement. These measures enhance SDM effectiveness in pediatric care and advance research in this field.

### 4.6 Integration of SDM with precision medicine

In this study, we integrated SDM with precision medicine to enhance treatment outcomes and reduce side effects through personalized care. The SDM model focused on the needs of pediatric patients and their families and incorporated assessments of disease characteristics, genetic information, and living environments. For instance, anti-inflammatory medications were tailored based on genetic testing results for specific gene mutations causing recurrent pneumonia. Rehabilitation plans were customized according to each child’s condition, with less stringent plans for milder cases and more rigorous measures for complex situations. Transparency was ensured by explaining these data analysis methods to patients and their families, fostering trust and active participation. Additionally, monitoring biomarkers such as inflammatory factors allowed for timely adjustments to treatment plans. Integrating these biomarker data into decision-making checklists enhanced treatment specificity and provided valuable data for future research.

## 5 Conclusion

This study demonstrates that SDM significantly enhances transition readiness, improves quality of life, in children with recurrent pneumonia. SDM involves patients and their families in the care process, aligning with the principles of precision medicine by offering tailored healthcare solutions that meet individual needs. The findings emphasize the importance of patient-centered approaches in pediatric care, showcasing the potential of SDM to optimize treatment outcomes and enhance overall patient satisfaction. As healthcare continues to evolve, integrating SDM into routine practice will be essential for delivering high-quality, personalized care to pediatric patients.

## Data Availability

The original contributions presented in this study are included in this article/supplementary material, further inquiries can be directed to the corresponding author.
